# Factors Influencing Intracavitary Electrocardiographic P-Wave Changes during Central Venous Catheter Placement

**DOI:** 10.1371/journal.pone.0124846

**Published:** 2015-04-27

**Authors:** Guorong Wang, Ling Guo, Bin Jiang, Min Huang, Jian Zhang, Ying Qin

**Affiliations:** 1 Department of nursing, Sichuan Cancer Hospital, Chengdu, P. R. China; 2 Central cenous catheterization room, Sichuan Cancer Hospital, Chengdu, P. R. China; 3 Department of thoracic surgery, Sichuan Cancer Hospital, Chengdu, P. R. China; 4 Department of radiotherapy, Sichuan Cancer Hospital, Chengdu, P. R. China; 5 Department of medical oncology, Sichuan Cancer Hospital, Chengdu, P. R. China; Bambino Gesù Children's Hospital, ITALY

## Abstract

Amplitude changes in the P-wave of intracavitary electrocardiography have been used to assess the tip placement of central venous catheters. The research assessed the sensitivity and specificity of this sign in comparison with standard radiographic techniques for tip location, focusing on factors influencing its clinical utility. Both intracavitary electrocardiography guided tip location and X-ray positioning were used to verify catheter tip locations in patients undergoing central venous catheter insertion. Intracavitary electrocardiograms from 1119 patients (of a total 1160 subjects) showed specific amplitude changes in the P-wave. As the results show, compared with X-ray positioning, the sensitivity of electrocardiography-guided tip location was 97.3%, with false negative rate of 2.7%; the specificity was 1, with false positive rate of zero. Univariate analyses indicated that features including age, gender, height, body weight, and heart rate have no statistically significant influence on P-wave amplitude changes (P>0.05). Multivariate logistic regression revealed that catheter insertion routes (OR = 2.280, P = 0.003) and basal P-wave amplitude (OR = 0.553, P = 0.003) have statistically significant impacts on P-wave amplitude changes. As a reliable indicator of tip location, amplitude change in the P-wave has proved of good sensitivity and excellent specificity, and the minor, zero, false positive rate supports the clinical utility of this technique in early recognition of malpositioned tips. A better sensitivity was achieved in placement of centrally inserted central catheters (CICCs) than that of peripherally inserted central catheters (PICCs). In clinical practice, a combination of intracavitary electrocardiography, ultrasonic inspection and the anthropometric measurement method would further improve the accuracy.

## Introduction

Central venous access, including centrally inserted central catheters (CICCs) and peripherally inserted central catheters (PICCs) placement, is widely used in the clinical management of cancer patients, and is frequently a necessity for the delivery of chemotherapy, for surgical operations and for critical care. Correct catheter tip location is crucial for the safe use of the central venous catheters (CVC) and for the prevention of complications. The Infusion Nurses Society (INS) recommends a post-insertion chest X-ray to check the CVC tip position. This technique, however, cannot be used to identify abnormal tip positions during the process of catheter insertion, and it is not always feasible, for patients who are in the immediate post-operative state, or in critical conditions. In addition, the X-ray inspection increases the exposure of patients to radiation.

Intracavitary electrocardiography(ECG) guided tip positioning has been introduced since 1980’s. Studies indicated that the CVC entering superior vena cava induced changes in the P-wave, typically including increased amplitude, notching of the P-wave (also described as an ‘M-shaped’ P-wave), or a dual-phase P-wave. The changes in the P-wave are typically seen when the catheter tip reaches the superior vena cava at the right atrial junction [1–3]. These changes can therefore be used as an indicator of the position of the CVC tip as it enters the superior vena cava, enabling a real-time awareness of catheter position, instant correction of malpositions, and better CVC positioning technique.

Despite this, some studies have found that the specific P-wave changes are not always observed in all patients during CVC insertion. The success rate of tip positioning during intracavitary electrocardiography-guided CVC insertion ranged from 76% to 97% [1,4]. In one study of catheter insertion via the internal jugular vein, the P-wave showed a larger amplitude and wider peak, with dual-phase and notched P-waves also seen as typical changes. Using any one or both changes as an indicator of position, the sensitivity of this technique for determining the correct position reached 97.7% [3,4]. We therefore aimed to study P-wave changes during intracavitary electrocardiography guided tip location as well as to determine the factors that might influence them, so as to clarify the indications for this technique and to determine its clinical utility.

## Subjects and Methods

### 1.1 Subject recruitment and exclusion criteria

The study objects were cancer patients undergoing central venous catheter placement in the CVC department of Sichuan Cancer Hospital from September 2013 to April 2014. Eligibility required age ≥16 years, confirmation of sinus rhythm by the doctor in charge of central venous access; and planned access via PICC, or CICC using the internal jugular vein. The study has been approved by the Ethics Committee of Sichuan Cancer Hospital. Informed written consent was obtained from all subjects. Excluded were patients who had one or more of the following conditions: 1) patients not to undergo PICC or CICC placement for a change in access plan; 2) patients with a cardiac pacemaker; 3) patients with dynamic electrocardiography equipment; and 4) patients whose testing imaging showed unstable basal ECG or intracavitary ECG; unidentifiable p-wave and/or QRS-wave.

A total of 1200 patients consented to participate in the study, age ranging from 18 to 91 years old. The final number of subjects achieving standards of enrollment were 1160, as 9 subjects withdrew from CVC insertion before the procedure, while 31 subjects were excluded for conditions list above.

### 1.2 Materials and Methods

#### 1.2.1 Materials

The ultrasonic instrument used to guide the puncture was the GE portable ultrasonic apparatus. The intracavitary (IC) electrocardiography monitor used was the Mindary iMEC8 monitor with electrocardiac signal amplifier and lead wires from Braun, Germany. The venous access devices were classified as follows: short term central venous catheters (Braun, Germany) and peripherally inserted central catheters (Argon, USA; Bard Groshong, USA). For the procedure, 2% lidocaine hydrochloride and 10 U/ml heparin sodium saline were used.

#### 1.2.2 Access team

A doctor and a nurse who had been trained for in CVC insertion prior the study were responsible for catheter insertion. One assistant nurse took charge of the connection and recording of intracavitary electrocardiography.

#### 1.2.3 Operational procedure

The catheters were inserted according to the internal jugular vein placement protocol and PICC insertion protocol. The electrocardiography IC connection was set up when catheter was expected to enter the superior vena cava. Posteroanterior chest X-ray was performed after catheter placement. Once there was suspicious or unsatisfactory X-ray result, alternative means were taken to confirm that the tip location, e.g. a lateral view. The tip positions for all subjects was commented on by the same radiologist, with catheter direction and tip location of each subject reviewed and recorded by nurses trained in specific.

#### 1.2.4 Lead connection for ECG monitoring

The intracavitary electrocardiography lead connection was set up by connecting one end of the lead wire to electrocardiography monitor via an adaptor; the other end connected to the external end of the supporting guide wire via a metal clip.

#### 1.2.5 ECG monitoring

The amplification ratio of the ECG wave amplitude was set to 1times, and lead II was selected to monitor the electrocardiac signal. Prior to CVC insertion, the patient was in the supine position for recording of a baseline surface ECG and a desired placement length. The desired length of catheter is predicted by measuring the distance from the puncture point to the sternoclavicular joints plus the distance from the sternoclavicular joints to the 3rd intercostal space on the right parasternal line. After the expected length was reached, CVC advancement took a pause and the lead connections were secured; Intracavitary electrocardiogram was then printed. When the catheter tip reach the expected length but no P wave amplitude change or just some disturbed waves were observed, the connection should be adjusted and re-established, and the catheter should be drawn-back and re-attempt for no more than three times.

### 1.3 Observation criteria and result interpretation

The criteria for proper identification of tip location on chest X-ray include: radiological landmark of the lower 1/3 of SVC, under the carina but within the first distal 3 cm. The change in amplitude of the P-wave on intracavitary electrocardiogram was used as the indicator for tip location at the target position. The correlation between tip location by intracavitary electrocardiography and by X-ray check was analyzed to explore factors that might influence changes in the amplitude of the P-wave.

#### 1.3.1 Amplitude of P-wave

The amplitude was defined as the distance between the P-wave peak position and ECG baseline, with the unit of millivolt (mV).

#### 1.3.2 Change in amplitude of P-wave

The change in P-wave amplitude was defined as ≥1 mV changes of the absolute value of the P-wave amplitude on intracavitary electrocardiogram (including dual-phase P-waves, and notched P-waves) compared to baseline P-wave amplitude [4,5].

#### 1.4 Statistical methods

A SPSS 13.0 software package was used for statistical analysis. The t-test and chi-squared test were used for univariate analysis, and logistic regression was used for multivariate analysis.

## Results

### 2.1 Patients’ characteristics

As shown in Table 1, of the 1160 subjects, 592 subjects were male (51.0%), and 568 were female (49.0%). The age range was 18–91 years old. As shown in Table 2, a total of 873 subjects (75.3%) received internal jugular vein CICC placement and 287 received PICC placement (24.7%). the range of amplitude changes in the P-wave were -2 mV to 40 mV, with an average value of 3.77 ± 2.89 mV, and P_50_ of 3 mV.

**Table 1:** Patients’ characteristics and ECG results of the subjects ($\bar{x}$ ± S, n = 1160).

	Age(year)	Height(cm)	Body weight(kg)	Heart rate(beats/min)	P-wave amplitude(mV)
$\bar{x}$ ± S	54 ± 11.59	160 ± 7.32	57.56 ± 9.69	81.46 ± 14.47	0.93 ± 0.69

**Table 2:** Catheter placements and P-wave amplitude changes for the subjects (n = 1160).

	CICC		PICC		Changes in P-wave amplitude	
	Left	Right	Left	Right	Yes	No
Cases	73	800	178	109	1119	41
%	6.3	69.0	15.3	9.4	96.5	3.5

### 2.2 Comparison of tip location by intracavitary electrocardiography and by chest X-ray

When the chest X-ray was used as the standard method for CVC tip location, the sensitivity of electrocardiography guided tip positioning accounted 97.3%; the specificity 1, with a false negative rate of 2.7% and a false positive rate of zero. The crude agreement rate was 97.3% (as shown in Table 3).

**Table 3:** Comparison of tip locations by intracavitary electrocardiography and chest X-ray (n = 1160).

Intracavitary electrocardiography	Chest X-ray		Sum
	+	-	
+	1119	0	1119
-	31	10	41
Sum	1150	10	1160

### 2.3 Correlation analysis of patient-specific charateristics, catheter placement and changes in P-wave amplitude of intracavitary electrocardiography

It is revealed that patients’ basal P-wave amplitude, catheter insertion routes, and puncture sites have significant impact on the scale of changes in P-wave amplitude (Table 4).

**Table 4:** Factors influencing changes in P-wave amplitude on intracavitary electrocardiography (n = 1160).

Factors	P-wave amplitude changes		Statistics	P value
	Yes	No		
Age	54.03 ± 11.60	53.02 ± 11.38	t = 0.546	0.585
Height	160.29 ± 7.31	159.24 ± 7.46	t = -0.898	0.370
Body weight	57.48 ± 9.72	59.63 ± 8.79	t = 1.399	0.162
Gender				
Male	572 (94.4)	20 (5.6)	χ^2^ = 0.086	0.769
Female	547 (92.0)	21 (8.0)		
Heart rate	81.46 ± 14.56	81.44 ± 12.36	t = -0.010	0.992
P-wave amplitude	0.92 ± 0.67	1.32 ± 1.01	t = 3.673	0.016*
Catheter insertion site				
Left	170 (90.2)	12 (9.8)	χ^2^ = 5.924	0.015
Right	949 (93.8)	29 (6.2)		
Catheter types				
CICC	851 (94.1)	22 (5.9)	χ^2^ = 10.650	0.001
PICC	268 (90.8)	19 (9.2)		

* Heterogeneity of variance

### 2.4 Non-conditional logistic regression analysis for factors influencing amplitude changes in the P-wave

Non-conditional logistic regression analysis was performed to analyze whether basal P-wave amplitude, catheter insertion routes, and puncture sites are independent factors that affect the P-wave amplitude change. The multivariate analysis, using a test level of α = 0.05, indicated that catheter insertion routes and the basal P-wave amplitude had statistically significant impacts on amplitude of P-wave during CVC placement (Table 5).

**Table 5:** Multivariate logistic regression analysis of P-wave stability in intracavitary electrocardiography (n = 1160).

Items	β	S.E.	Wald	P	OR	95%CI	
Constant terms	2.592	0.639	16.465	0.000	13.353		
Insertion route	0.824	0.331	6.206	0.013	2.280	1.192	4.359
P-wave amplitude	-0.593	0.200	8.745	0.003	0.553	0.373	0.819

## Discussion

Earlier papers regarded trans-esophageal echocardiography(TEE) as a most accurate method for tip location, and quite a few authors have evaluated the accuracy of the IC-ECG method in comparison with X-ray [1,2,4–6]. One fact is that choosing X-ray rather than TEE is in accordance with the guide by the Infusion Nursing Standards of Practice by INS (2011) in which chest X-ray is a must for tip confirmation after placement [7]. In China, according to the Chinese version of Infusion Nursing Standards of Practice guideline, a post-procedure radiological confirmation should be carried out after placement for patients’ safety. In addition, in clinical practice, X-ray is more cost-effective than TEE and remains the most common method for most VADs.

Considering that the ideal position for the tip of the catheter is restricted in the lower 1/3 of the superior vena cava or at the cavo-atrial junction [7], accurate positioning is of great importance. The conductivity of the catheter guide wire and blood can be used to set up an intracavitary electrocardiogram, thus providing specific P-wave changes to identify catheter tip location. The use of intracavitary electrocardiography, apart from the commonly used post-insertion X-ray check, is becoming increasingly popular for assessment of the catheter placement accuracy. Some studies even suggested for replacing X-ray check with intracavitary electrocardiography as the new standard for central catheter tip location [1]. Yet other studies, in contrast, found that in a number of cases (3.8%), the results from intracavitary electrocardiography had insufficient correlation with X-ray positioning, while in a minority of cases (0.7%) P-wave amplitude changes were not observed at all [2].

In this study we compared the tip location results from intracavitary electrocardiography-supported placement with the results of X-ray examination, and found there are 1129 cases out of 1160 cases obtained consistent results from both examine approaches. Taking X-ray results as standard, intracavitary electrocardiograph has a sensitivity rate of 97.3%, a specificity rate of 100%, and a false positive rate of zero. Furthermore, all the suspected cases judged as malpositioned were first detected by intracavitary electrocardiography, then confirmed and corrected by ultrasonic assessment. No false positive case was observed. In this sense, tip location by intracavitary electrocardiography has significant advantages in clinic use, especially for early detection of errors in CICC and PICC tip location.

In the current study, 31 cases with proper placement showed no specific changes in P-wave amplitude. The sensitivity was lower than that as reported by Pitti et al [2]. The reasons could lie in the differences in insertion routes and/or the lead connection devices used. In addition, 31 cases were excluded from this study due to failure in obtaining satisfactory electrocardiogram. Improved lead connection and application of additional techniques such as ultrasonic may result in more accurate tip positioning, thus enhance the application value of intracavitary electrocardiograph in clinical practice [8].

In the current study, based on 1160 cases performed intracavitary electrocardiography-guided tip location, we explored the factors that might have impact on P-wave amplitude change. The results showed that age, gender, height, body weight and heart rate had no statistically significant association with P-wave amplitude changes (P>0.05), indicating that intracavitary electrocardiography guided tip location has good applicability to varied patient-specific conditions. Kremser et al. [9] suggested that the accuracy of intracavitary electrocardiography guided tip location was relatively low in cases of left-sided CVC placement, for which an emendation was recommended. In our study, P-wave amplitude changes were seen in 90.2% of left-sided insertion cases, but no statistically significant differences between left-sided and right-sided insertion were found in multivariate logistic regression analysis. We therefore propose that intracavitary electrocardiography-guided tip location can be used for left-sided CVC insertion as long as the quality of electrocardiographic connection is ensured.

As to the effects of basal P-wave amplitude, Moureau et al. [1] excluded subjects with indistinguishable P-wave shapes at the time of case selection. In the current study, however, patients with low basal P-wave amplitudes inversely have higher P-wave amplitude changes (OR = 0.553, P = 0.003). We thus conclude that patients with high P-wave amplitude in the surface electrocardiogram do not necessarily present typical P-wave change in intracavitary electrocardiography. Patients with low basal P-wave amplitude should still be considered as candidates for this technique.

Results in the present study showed that clinically useful P-wave amplitude changes were more readily found in CICC placement than in PICC placement. We suggest that the variation might be related to the differences in the catheters. Shorter, larger catheter provides more stable lead connection/signal transductions in CICC placement compared with PICC placement. The CICC guide wire used in this study consists of multiple metal strands and the diameter of CICC measures 0.8 mm, in contrast to the ≤0.5mm diameter of the PICC. Better sensitivity was then achieved in CICC placement in intracavitary electrocardiography-guided tip location. We believe that by applying specially designed ECG devices with better lead connection and improved signal transduction, the applicability of this technique will be further enhanced in clinical practice.
